# Dosiseskalation bei der Radiotherapie inoperabler Ewing-Tumoren

**DOI:** 10.1007/s00066-023-02046-0

**Published:** 2023-01-17

**Authors:** Constanze Polzer, Jürgen Dunst

**Affiliations:** Klinik für Strahlentherapie Kiel, UKSH, Kiel, Deutschland

## Hintergrund und Ziele

Das Ewing-Sarkom (heute: Gruppe der Ewing-Tumoren) wird seit seiner Erstbeschreibung zwar als strahlenempfindlicher Tumor angesehen; dennoch gilt die Operation bei R0-resektablen Tumoren als lokale Therapie der ersten Wahl [1, 2]. Die klassische, in zahlreichen prospektiven Studien verwendete Strahlendosis beträgt bei definitiver Therapie inoperabler Tumoren mindestens 45 Gy, meistens 54–60 Gy [9]. Trotz der teilweise unbefriedigenden lokalen Kontrolle gab es bisher keine Studien mit höheren Strahlendosen. In einer indischen Studie des Tata Memorial Center aus Mumbai wurde jetzt erstmals die Wirksamkeit einer Dosiserhöhung beim inoperablen Ewing-Sarkom auf 70,2 Gy untersucht [7].

## Patienten und Methoden

In die prospektive, randomisierte Studie schlossen die Autoren Kinder und junge Erwachsene ein, die ein histologisch bestätigtes, nicht metastasiertes extrakranielles Ewing-Sarkom/PNET hatten. Weiterhin musste dieses nach Induktionschemotherapie als nicht operativ entfernbar gelten. Untersucht wurde die Wirkung einer Dosiserhöhung bei der Strahlentherapie des inoperablen Ewing-Sarkoms. Zwischen April 2005 und Dezember 2015 wurden 99 Kinder und junge Erwachsene zwischen 13 und 23 Jahren in die Studie aufgenommen, 95 wurden randomisiert. Alle Patienten erhielten im Rahmen der Basisdiagnostik und zur Bestrahlungsplanung eine MRT der Tumorregion und eine FDG-PET-CT. Nach neunwöchiger Induktionschemotherapie erfolgte die Randomisierung in die Gruppen Standardstrahlendosis (SDRT, 55,8 Gy in 31 Fraktionen) versus Eskalationsdosis (EDRT, insgesamt 70,2 Gy in 39 Fraktionen, d. h. 55,8 Gy plus 14,4 Gy Boost); die Bestrahlung erfolgte in beiden Armen in konventioneller Fraktionierung (5 × 1,8 Gy pro Woche). Das mediane Tumorvolumen (der wichtigste Prognosefaktor) war sehr hoch, nämlich in der SDRT-Gruppe 289 cm^3^ und in der EDRT-Gruppe sogar 392 cm^3^; ferner waren 63 % der Tumoren im Becken lokalisiert. Die mediane Dauer der Strahlentherapie war 44 Tage im SDRT-Arm und 53 Tage im EDRT-Arm. Das Follow-up erfolgte bei beiden Gruppen im ersten Jahr alle drei Monate, die nächsten zwei Jahre alle sechs Monate, danach jährlich. Als primärer Endpunkt wurde die lokale Kontrolle gewählt, sekundäre Endpunkte waren das krankheitsfreie Überleben und das allgemeine Überleben.

## Ergebnisse

Alle Patienten beendeten die vorgesehene Strahlentherapie und wurden über einen medianen Zeitraum von 67 Monaten beobachtet, die primären und sekundären Endpunkte wurden für einen Zeitraum von fünf Jahren berechnet. In allen drei Endpunkten (lokale Tumorkontrolle, krankheitsfreies Überleben, Gesamtüberleben) zeigten sich bessere Ergebnisse für den dosiseskalierten Arm. Die lokale Tumorkontrolle nach 5 Jahren war signifikant höher im dosiseskalierten EDRT-Arm als im SDRT-Arm (76,4 % versus 49,4 %, *p* = 0,02), bezüglich das krankheitsfreien Überlebens (46,7 % versus 31,8 %, *p* = 0,22) und des Gesamtüberlebens nach 5 Jahren (58,8 % versus 45,4 %, *p* = 0,08) wurde allerdings formal keine Signifikanz erreicht. Zu einem Lokalrezidiv kam es bei 29,8 % (*n* = 14) der Patienten im SDRT-Arm gegenüber 6,3 % (*N* = 3) im EDRT-Arm. Fernmetastasen traten bei 23,4 % (*n* = 11) der Patienten in der Standarddosisgruppe auf gegenüber 35,4 % (*n* = 17) im EDRT-Arm. In der Subgruppenanalyse konnte kein signifikanter Unterschied in den einzelnen Subgruppen beobachtet werden, außer zwischen SDRT- und EDRT-Arm. Im EDRT-Arm gab es eine erhöhte Rate von akuten Hautreaktionen (10,4 % vs. 2,1 %, *p* = 0,08); die funktionellen Langzeitergebnisse waren in den beiden Armen gleich gut. Höhergradige Spätfolgen (Grad ≥ 3) wurden nur je eine in jeder Gruppe festgestellt, was die gute Durchführbarkeit der Strahlentherapie mit erhöhter Dosis unterstreicht.

## Schlussfolgerung der Autoren

Eine Erhöhung der Strahlendosis auf 70 Gy führt bei alleiniger Strahlentherapie inoperabler Ewing-Tumoren zu einer signifikanten Verbesserung der 5‑Jahres-Lokalkontrolle mit einem im Trend besseren Gesamtüberleben. Die Autoren empfehlen, die Möglichkeit der Dosiseskalation bei inoperablen Tumoren bei der Therapieplanung zu berücksichtigen.

## Kommentar

Die Heilungsraten bei Ewing-Tumoren haben sich in den 1970er- und 1980er-Jahren mit Einführung der Chemotherapie erheblich verbessert [1, 2, 4–6]. Die demgegenüber eher geringeren weiteren Verbesserungen der 1990er-Jahre sind (aus Sicht der Strahlentherapie) zum großen Teil auch durch optimierte Strahlentherapie entstanden, nämlich intensivierte Lokaltherapie (prä- oder postoperative Bestrahlung bei großen Tumoren), Ganzlungenbestrahlung bei pulmonaler Metastasierung und Radiotherapie von Knochenmetastasen als Ergänzung zur Hochdosistherapie bei primär extrapulmonaler Metastasierung [3]. In den letzten 20 Jahren gab es allerdings kaum weiteren Fortschritt in der Therapie der Ewing-Tumoren. Übersichtsarbeiten sehen mögliche zukünftige Verbesserungen vor allem durch neue Medikamente [4]. Dass es auch bezüglich der Strahlentherapie ein Optimierungspotenzial geben könnte, wird kaum beachtet. Diese Studie belegt eindrucksvoll das Gegenteil.

Warum kommt diese Studie erst jetzt? Die Dosis von 54 bis 60 Gy für inoperable Ewing-Tumoren wurde in den großen Studien der internationalen Studiengruppen vor mehr als 20 Jahren als optimal identifiziert und danach einfach beibehalten. Die Dosierungskonzepte der Strahlentherapie stammen also aus der 2‑D- oder frühen 3‑D-Ära und sind wesentlich geprägt von der Furcht vor Spätfolgen in diesem sensiblen Kollektiv. Die meisten Studienaktivitäten konzentrierten sich in den letzten Jahren und auch aktuell auf Fragestellungen um die Systemtherapie. Dass sich eine europäische oder US-Studiengruppe auf eine strahlentherapeutische Fragestellung bei Ewing-Tumoren einigt, wie sie jetzt in der am Tata Memorial Center in Mumbai durchgeführten Studie untersucht wurde, ist ziemlich unwahrscheinlich. Man kann den indischen Kollegen also nur gratulieren. Nebenbei: Das Tata Memorial Center, das größte und renommierteste Cancer Center in Indien, hat schon durch andere Studien (z. B. POP-RT beim Prostata-Ca) auf sich aufmerksam gemacht, und wir Europäer und US-Amerikaner müssen uns sicher daran gewöhnen, dass wichtige neue Daten vor allem in der pädiatrischen Onkologie zukünftig aus Indien kommen werden. Und als Radioonkologen können wir dankbar sein, dass die Strahlentherapie an den indischen Krebszentren einen ungewöhnlich hohen Stellenwert hat. Folgende Punkte sind aus unserer Sicht beachtenswert:

- Die hier beobachtete Lokalrezidivrate von 35 % im SDRT-Arm ist im internationalen Vergleich sehr hoch. Dies ist aber durch die ungünstige Patientenselektion erklärbar. Im dosiseskalierten EBRT-Arm der Studie wurden in diesem Risikokollektiv lokale Kontrollraten erreicht wie bei Resektion mit oder ohne Strahlentherapie.
- Ein wichtiger Aspekt betrifft die Tatsache, dass trotz der erhöhten Dosis mit Boost von 14 Gy keine signifikanten Unterschiede in der Langzeittoxizität und Funktionalität im Vergleich zur Standarddosis zu erkennen waren. Das spricht dafür, dass eine mit modernen RT-Techniken durchgeführte moderate Dosiseskalation gut vertretbar ist. Die leicht erhöhte Rate an akuten Hautreaktionen ist aus unserer Sicht gut vertretbar vor dem Hintergrund der verbesserten Tumorkontrolle.
- Dass mit einem so kleinen Kollektiv (*N* = 99) überhaupt in irgendeinem relevanten Endpunkt ein signifikantes Ergebnis erzielt wurde, ist ungewöhnlich. Die meisten randomisierten Studien der letzten 20 Jahre zeigten auch in großen Kollektiven nur marginale, selten signifikante Verbesserungen. Der Unterschied in der Gesamtüberlebensrate in dieser Studie ist mit über 10 %-Punkten (59 % vs. 46 %) erheblich und sollte, wenngleich mit *p* = 0,08 formal nicht signifikant, beachtet werden.
- Der Vorteil durch die erhöhte Dosis von 70 Gy ist in dieser Studie so hoch, dass man die Frage stellen darf, ob eine solche optimierte Strahlentherapie vielleicht nicht nur bei inoperablen, sondern auch bei operablen Tumoren sinnvoll ist, denn die Überlebensrate von fast 60 % im EDRT-Arm ist für dieses High-risk-Kollektiv sehr gut.

Aus unserer Sicht ist diese Studie beachtlich, weil sie festgefahrene und aus heutiger Sicht suboptimale Therapiekonzepte hinterfragt und das Potenzial der modernen Strahlentherapie aufzeigt. Diese Studie reicht sicher nicht aus, um zukünftig jedes Kind mit einem Ewing-Tumor mit 70 Gy zu bestrahlen. Sie ist aber bestens geeignet, eine interdisziplinäre Diskussion über den Stellenwert der Lokaltherapie anzustoßen und auf weitere Potenziale der Strahlentherapie, z. B. einen möglichst frühen Zeitpunkt der Bestrahlung, hinzuweisen; auch bezüglich des Timings besteht nämlich aus unserer Sicht noch erhebliches Optimierungspotenzial [3, 8].

## Fazit

Diese indische Studie von Laskar und Mitarbeitern zeigt, dass eine moderate Dosiseskalation auf 70 Gy zu einer signifikant besseren lokalen Tumorkontrolle und im Trend auch zu deutlich besseren Überlebensraten bei inoperablen Ewing-Tumoren führt, und das ohne erhöhte Spättoxizität. Die Studie zeigt das Potenzial der modernen Strahlentherapie in der pädiatrischen Onkologie, und auch wenn man aus dieser Studie keine generelle Empfehlung für höhere Strahlendosen ableiten kann, sollte man die Daten im Einzelfall berücksichtigen und in die interdisziplinäre Diskussion und radioonkologische Therapieplanung einbringen.

*Constanze Polzer und Jürgen Dunst, Kiel*
